# The effects of a 6-month Schroth intervention for Adolescent Idiopathic Scoliosis (AIS): preliminary analysis of an ongoing randomized controlled trial

**DOI:** 10.1186/1748-7161-8-S2-O44

**Published:** 2013-09-18

**Authors:** Sanja Schreiber, Eric C Parent, Douglas M Hedden, Marc Moreau, Douglas Hill, Elise M Watkins

**Affiliations:** 1 University of Alberta, Faculty of Rehabilitation Medicine, Alberta, Canada

## Background

Literature lacks strong evidence on the effect of exercises on scoliosis[1]. Schroth scoliosis-specific exercises have shown promising results in studies of suboptimal quality.

## Purpose

The purpose of this study was to evaluate the effect of Schroth exercises on back endurance in patients with AIS using the Scoliosis Research Society-22r (SRS-22r) and Self-Efficacy (SEQ) questionnaires. 

## Methods

A total of 31 patients with AIS, aged 10-18, with curves from 10°-45°, wearing a brace or not, participated. Sixteen were randomized to Schroth with standard care, and 15 to standard care alone (monitoring or bracing) for six months. The Schroth intervention consisted of five individual visits to learn the exercises, followed by weekly supervised group sessions of one hour each, with daily home exercises prescribed using an algorithm [2] (45 minutes per day). Compliance was monitored with a logbook, and outcomes were recorded at baseline and six months. Effect sizes were estimated using Cohen’s d, which corresponds to the mean difference between the groups in the change observed from baseline to six months (Schroth – standard care), divided by the pooled standard deviation at baseline (Cohen’s d ≥0.8=large, 0.5-0.8=moderate, 0.2-0.5=small[3]).

## Results

Two controls and one Schroth group participant dropped out. Mean age was 14.4±2.1yrs for Schroth and 13.7±1.7yrs for controls; mean Cobb angles were 32.6±7.9o and 28.8±10.0o, respectively. Schroth participants with complete follow-up attended 87±8% of the prescribed weekly exercise sessions and completed 86±5% of the prescribed home exercises. Intention-to-treat analysis lowered compliance to 83±19% and 81±17% for weekly sessions and home program, respectively. Effect sizes at six months for the SRS-22r were smaller than expected, but favored the Schroth group with Cohen’s d: pain=0.09, self-image=0.09, function=0.00 and total=0.21. The effect sizes for self-efficacy (0.18) and for the Biering-Sorensen test (0.28) also favored Schroth. The perceived mean global rating of change in the Schroth group was 3.8±2.2, corresponding to moderate improvement, and -0.3±1.7 in the standard care group, corresponding to a small amount of deterioration. 

## Conclusions and discussion

The dropout rate was low (9.7%), which was reflective of the patients’ commitment to the therapy. Outcomes favored the Schroth group. In this preliminary analysis, Schroth exercises showed a small but positive influence on self-efficacy, self-image, pain and back muscle endurance. 
